# Influence of mycorrhizae and silicon on maize and soybean resistance and tolerance to *Spodoptera frugiperda* (Lepidoptera: Noctuidae)

**DOI:** 10.1093/jisesa/ieag028

**Published:** 2026-03-29

**Authors:** Lucas Adjuto Ulhoa, Arlindo Leal Boiça Júnior, Jeffrey A Davis, Michael Joseph Stout

**Affiliations:** Departamento de Ciências da Produção Agrícola, Universidade Estadual Paulista, Jaboticabal, Brazil; Departamento de Ciências da Produção Agrícola, Universidade Estadual Paulista, Jaboticabal, Brazil; Department of Entomology, Life Sciences Building, Louisiana State University, Baton Rouge, LA, USA; Department of Entomology, Life Sciences Building, Louisiana State University, Baton Rouge, LA, USA

**Keywords:** induced plant defense, host plant resistance, soil amendment, root symbiont, sustainable crop protection

## Abstract

Productivity of soybean, *Glycine max* (L.) Merr. (Fabales: Fabaceae), and maize, *Zea mays* L. (Poales: Poaceae), is reduced by several insect pests, among the most important of which is the fall armyworm, *Spodoptera frugiperda* (J.E. Smith) (Lepidoptera: Noctuidae). Control of this insect relies mainly on chemical insecticides or transgenic plants expressing *Bacillus thuringiensis* Berliner (Eubacteriales: Bacillaceae) proteins, but these methods impose strong selection pressure favoring resistance evolution. Soil applications of silicon (Si) and arbuscular mycorrhizal fungi (AMF) are potential alternative tactics to enhance plant resistance and tolerance within integrated pest management programs. This work evaluated the effects of soil application of Si and AMF, individually and in combination, on the development of soybean and maize and on their resistance and tolerance to *S. frugiperda*. Greenhouse experiments used 4 treatments: control, Si application, AMF inoculation with *Rhizophagus irregularis* (Błaszk., Wubet, Renker & Buscot) (Glomerales: Glomeraceae), and combined Si + AMF. AMF inoculation significantly increased root and shoot biomass of soybean and plant height in maize, while Si significantly increased root biomass in maize. The combined AMF + Si treatment significantly reduced defoliation by *S. frugiperda* on soybean. AMF-inoculated soybean plants also recovered better from mechanical defoliation, producing greater shoot biomass, whereas Si-treated maize showed higher root biomass after injury. These findings demonstrate that Si and AMF soil applications can enhance crop development and contribute to resistance and tolerance against fall armyworm, offering sustainable alternatives for integrated pest management in soybean and maize.

## Introduction

Soybean, *Glycine max* (L.) Merr. (Fabales: Fabaceae), and maize, *Zea mays* L. (Poales: Poaceae), are 2 of the most widely cultivated crops in the world, being sources of protein, oil, and carbohydrates for both human and animal consumption. In addition, these crops are used in the production of pharmaceuticals, biofuels, cosmetics, oil, ethanol, and plastic. However, maize and soybean productivity are negatively affected by the presence of several insect pests, among the most important of which is the fall armyworm, *Spodoptera frugiperda* (J.E. Smith) (Lepidoptera: Noctuidae) (Hoffmann-Campo et al. 2012, Quintela and Barbosa 2015, Valicente 2015, Eghrari et al. 2022). This lepidopteran injures all maize development stages and can cause yield reductions of up to 60% (Barros et al. 2010, Valicente 2015). Larvae of *S. frugiperda* are also a major threat in soybean-producing countries like Brazil and the United States. This is because it reproduces quickly in crops and can cause crop losses greater than 70% (Overton et al. 2021).

The most common tactic for management of this pest in the field is the application of chemical insecticides or plant incorporated protectants via insecticidal proteins from *Bacillus thuringiensis* Berliner (Eubacteriales: Bacillaceae) (Miraldo et al. 2016, Parisi et al. 2016, Chaudhary and Singh 2019). However, populations of this pest have evolved resistance to both chemical insecticides and Bt proteins in several regions (Gutiérrez-Moreno et al. 2019). In addition, *S. frugiperda* is difficult to control in maize, as this caterpillar lodges inside the whorl of the plant, making contact with insecticides difficult. In addition, several problems arise from the use of chemical insecticides, especially those related to human health (Li 2018), environmental impact (Tudi et al. 2021), and nontarget effects on natural enemies such as predators, parasitoids, and entomopathogenic fungi (Geiger et al. 2010), which act as biological control agents in the field (Bueno et al. 2017).

For these reasons, the search for alternative tactics for integrated pest management programs for fall armyworms is important. Two potential alternatives are applications of arbuscular mycorrhizal fungi (AMF) and silicon (Si) to the soil to enhance plant development and augment host plant resistance (Debona et al. 2017, Alhousari and Greger 2018, Higo et al. 2019, Schoenherr et al. 2019).

AMF are essential for many agricultural crops (Xiao et al. 2019). These fungi form mutualistic associations with crop roots aiding in nutrient and water absorption. In exchange, AMFs receive carbon assimilated by photosynthesis (Currie et al. 2011, Barber et al. 2013, Luginbuehl et al. 2017). With greater absorption of nutrients and water, plants can increase growth and better resist or tolerate abiotic (Abdel-Latef et al. 2016, Moradtalab et al. 2019) and biotic stresses, including injury to the roots and aerial portions of the plant (Gange 2001, Pozo and Azcon-Aguilar 2007, Smith and Read 2008, Vannette and Hunter 2011, Campos-Soriano et al. 2012, Schoenherr et al. 2019). In addition, AMFs can alter the secondary metabolism of plants (Hohnjec et al. 2005, Walker et al. 2012), such as increasing concentrations of endogenous hormones that can influence the feeding and development of herbivorous insects (Nishida et al. 2010, Tomczak et al. 2016), and altering the concentration and composition of terpenoids emitted by plants that alter plant attractiveness and insect behavior (Copetta et al. 2006, Khaosaad et al. 2006, Kapoor et al. 2007, Rapparini et al. 2008, Schausberger et al. 2012, Babikova et al. 2014, Shrivastava et al. 2015, Ulhoa et al. 2020).

Si is an abundant element which plants absorb as monosilicic acid (H_4_SiO_4_) (Paye et al. 2018). Upon reaching the intercellular spaces and cell walls of the plant epidermis, absorbed Si is polymerized and deposited as amorphous silica (SiO_2_  *n*H_2_O) (Meena et al. 2014). As a result, a mechanical barrier is formed (Massey and Hartley 2009), which makes plant tissues tougher and makes them difficult for herbivores to ingest and digest (Alhousari and Greger 2018). In addition, absorbed monosilicic acid can induce plant defenses through physiological and biochemical processes (Ma 2004, Liang et al. 2015, Singh et al. 2022). Si, like AMF, can trigger the production of phytohormones that promote resistance and emission of volatile compounds that repel herbivorous insects and attract their natural enemies (Gomes et al. 2005, Ranger et al. 2009, Kvedaras et al. 2010, Peixoto et al. 2011, Reynolds et al. 2016).

Despite the well-documented effects of Si and mycorrhizae separately in crops, there are only a few studies on the interactive effects of these 2 factors, particularly in maize and soybean (Ulhoa et al. 2025). The combined application of Si and AMF may provide synergistic effects for enhancing plant resistance against herbivorous insects like *S. frugiperda*. While AMF primarily enhance nutrient uptake and induce chemical defenses, Si provides physical barriers and can also trigger chemical defense pathways. Understanding these potential interactive effects is crucial for developing effective, sustainable pest management strategies that reduce reliance on chemical insecticides. In view of this, the objectives of this work were to evaluate the effects of the application of AMFs and Si in the soil, individually and in combination, on the development of maize and soybean and on the resistance and tolerance of these plants to *S. frugiperda* in the greenhouse.

## Materials and Methods

The experiments were conducted in a greenhouse. The temperature, relative humidity (RH), and photoperiod of the greenhouse were 35 ± 1 °C; 60 ± 10% RH and 12:12 L:D, respectively.

### Plants

Soybean cultivar UA5414RR (University of Arkansas System Division of Agriculture, Fayetteville, Arkansas, United States) and maize cultivar Silver Queen Hybrid (Burpee Seeds, Warminster, Pennsylvania, United States) were tested in these experiments. Plants were grown in 2 l polyethylene pots containing topsoil, sand, and peat in a ratio of 2:1:1, respectively. The topsoil was a sandy loam collected from a local agricultural field. No chemical analysis of the substrate was performed prior to the experiment. Neither the soil mixture nor the seeds were sterilized before use, as the goal was to simulate conditions more representative of field settings. Three soybean or maize seeds were sown per pot. One week after emergence, plants were thinned to 1 plant of similar size per pot. Solid granular fertilizer with nitrogen, phosphorus, and potassium (NPK 5-20-20) was applied by topdressing 2 wk after planting at a rate of 2 g per pot, following general recommendations for greenhouse-grown plants. This formulation was chosen to provide a balanced nutrient supply, particularly phosphorus and potassium, which are important for root development and overall plant health. Plants were watered every 2 d with tap water.

### Insects

The *S. frugiperda* caterpillars used in experiments came from a colony maintained in a laboratory. To maintain genetic variability and colony vigor, *S. frugiperda* caterpillars were collected annually from rice fields and therefore likely represent the rice strain (sfR) of this species. The insects were reared in a climate-controlled room with a temperature of 28 ± 2 °C, RH of 70 ± 10% and photoperiod of 14:10 L:D. The diet used to rear the caterpillars was a commercial formulation prepared specifically for this species (Southland Products Incorporated, Lake Village, Arkansas, United States). Although the exact composition is proprietary, the diet is a commercially prepared formulation designed specifically for rearing *S. frugiperda*, likely containing a combination of protein sources, vitamins, antimicrobials, and agar as a solidifying agent, as is typical of lepidopteran artificial diets. Upon reaching the pupal stage, pupae were transferred to 15-l buckets covered with gauze fabric. Buckets contained vermiculite at the bottom, cheesecloth gauze for an oviposition substrate, and 2 cotton dental rolls soaked in a solution containing 150 ml of honey, 150 ml of beer, 300 ml of water, and 12 g of ascorbic acid and covered with gauze. Egg masses were transferred daily to sealed 8-cell trays (Bio-Serv, Frenchtown, New Jersey, United States) containing moistened cotton. When the caterpillars hatched, they were placed individually into small plastic 30-ml cups containing artificial diet as previously described until used in the feeding trials in a greenhouse.

### AMF, Si, and Soil Amendments

A granular commercial inoculum (AMF-G) containing 300 propagules/g of the endomycorrhizal species *Rhizophagus irregularis* (Błaszk., Wubet, Renker & Buscot) (Glomerales: Glomeraceae) was used to promote symbiosis in soybean and maize roots (Premium Mycorrhizal Inoculant, WOW Wallace Organic Wonder, Coventry, Rhode Island, United States).

The Si source used for the greenhouse experiments was a commercial product containing wollastonite ore processed by crushing, drying, and grinding (Vansil W-10, Vanderbilt Minerals LLC, Norwalk, Connecticut, United States). The product granulometry was 19% at 200 mesh and 81% between 200 and 325 mesh. This product was a calcium silicate (Ca_2_SiO_4_) with a molar mass of 116.16 g/mol and contained 24% Si by mass.

A total of 6 separate experiments were conducted in the greenhouse, 3 with maize and 3 with soybean. In each of the 6 experiments, 60 potted plants were randomly assigned to 1 of 4 soil treatments (15 pots per treatment): (i) Control (no AMF-G or Si); (ii) Si; (iii) AMF-G; (iv) AMF-G + Si. The experimental design was completely randomized, with 4 treatments and 15 replications (pots with 1 plant) for both soybean and maize.

In pots assigned to Si treatments, 10 g of wollastonite (equivalent to 5 tons of Si/hectare) was applied in each pot. The wollastonite was placed on top of the soil after the soil was placed into the pots. After adding the wollastonite, all the pots were watered, and maize and soybeans were planted as described. In treatments with arbuscular mycorrhizal fungus, 8 g of granular inoculum of *R. irregularis* (AMF-G) was mixed into the soil before filling pots. An additional 1 g of AMF-G was added to the indentation in the soil into which seeds were placed. In this way, the seed was placed on top of the inoculant, ensuring the AMF-G came into contact with the roots of the seedlings when they germinated.

### Plant Growth and Development

Two experiments, one with maize and the other with soybean, were conducted to evaluate the individual and combined effects of AMF-G and Si on maize or soybean growth and development in undamaged plants. After growing plants in soil subjected to the 4 soil treatments described above, the following parameters of maize and soybean plants were measured: numbers of trifoliate leaves in soybean (TL), numbers of leaves in maize (NL), plant height (PH), root dry weight (R), and shoot dry weight (S). Plant height was measured from the soil surface to the tip of the highest fully extended leaf using a graduated ruler, with measurements recorded to the nearest 0.5 cm. Accordingly, plant height values are reported with 1 decimal place. NL and PH were measured at 15 and 30 d after planting (DAP) for maize and 30 and 45 DAP for soybean, whereas destructive measurements of root and shoot dry weights were made at 30 DAP for maize and 45 DAP for soybean. At all time points, 15 corn and soybean plants were evaluated. Dry weights were measured by carefully removing plants from pots, gently cleaning plants under running water, placing them in paper bags with the roots and shoots separated, and then drying plants in a 70 °C oven for 7 d. The plant parts were then weighed to the nearest 0.0001 g using a scale (Model Adventer AR2140, 2013, Ohaus, Florham, Park, New Jersey, United States). For presentation purposes, biomass values were rounded to 2 decimal places.

### Plant Recovery from Defoliation

In 2 additional experiments, 1 with maize and 1 with soybean, the impact of soil treatments on the recovery of plants following mechanical injury was evaluated. Each experiment followed the same completely randomized factorial design (2 × 2) with 15 replicates per treatment combination, as described above. For soybean, mechanical injury was imposed at 30 DAP. The injury was performed by cutting ∼80% of the leaf area using scissors, leaving only 20% of the foliage intact. This level of defoliation was visually estimated by a single trained individual, aiming to simulate the typical feeding damage caused by *S. frugiperda* larvae. While no physical template was used, care was taken to apply the injury uniformly across all treated plants. Numbers of trifoliate leaves on the soybean plants were counted before the injury was imposed. A similar experiment was conducted with maize plants, but for maize the mechanical injury was carried out at 15 DAP. Maize plants were injured by cutting the entire plant with scissors 5 cm from the soil line, leaving only stem tissue (no leaves).

For both maize and soybean plants, the recovery of the plants 15 d after injury was evaluated by counting the number of new soybean trifoliates or new maize leaves and measuring plant height and dry weights of the root and aerial portions. To weigh the plants (dry weight), the plants were carefully removed from the pots, then the plants were cleaned under running water, placed in paper bags, and dried for 7 d in a drying oven.

### *Spodoptera frugiperda* Growth and Development

To evaluate the effects of soil amendment with AMF and Si on defoliation and development of *S. frugiperda* caterpillars on maize and soybean, 2 experiments were conducted in which soybean and maize plants grown in soil treated with Si and AMF as described above were used for feeding assays with Fall Armyworm. Each experiment followed the same completely randomized factorial design (2 × 2) with 15 replicates per treatment combination.

Soybean plants were infested at 30 DAP and maize plants at 15 DAP. Before infestation, *S. frugiperda* caterpillars, ∼5 d old and corresponding to the third instar based on colony development timing, were starved for 3 h to void their guts and then weighed to obtain initial weights (IW). Instar determination was based on larval age and visual size under standardized rearing conditions; head capsule measurements were not performed. Although minor variability in instar assignment cannot be entirely excluded, the use of synchronized rearing under controlled environmental conditions minimized this potential source of variation. One third-instar caterpillar was placed with forceps on each plant, positioned above the newest fully emerged leaf. Caterpillars were allowed to feed for 6 d on soybean and 4 d on maize plants.

To confine insects to the plants, cylindrical cages made of polyethylene tubes measuring 30 cm in height and 10 cm in diameter were placed over each plant. The tops of these cages were covered with fine antiaphid screening mesh (mesh size ∼0.5 mm) to prevent caterpillar escape while allowing air circulation and light penetration. The cages were secured at the base around the pot to ensure a tight fit. Defoliation on soybean and maize plants was visually estimated by a single trained observer based on approximate classes of leaf area loss (≈5% increments). Mean defoliation values are therefore reported without decimal places to reflect the visual nature of the assessment. It should be noted that visual estimation is inherently subjective, and small differences among treatments may not be reliably detected without digital leaf area quantification methods. The caterpillars were weighed again, after another 3-h starvation period, to obtain the final weight (FW). The weight gain (WG), average weight (AW) and relative growth rate (RGR) of caterpillars were calculated. The RGR was calculated using the formula: (WG)/(AW×ND), where WG=FW−IW, AW=(FW+IW)/2 and ND (number of days the caterpillars fed on the plants).

### Quantification of Mycorrhizal Colonization on the Roots

To confirm that the roots of soybean and maize plants treated with AMF-G were colonized by *R. irregularis*, fungal structures (arbuscules and vesicles) were counted and observed under a microscope in the laboratory. Colonization was evaluated after experiments had been completed (45-day-old soybean plants and 30-day-old maize plants).

For root and fungus staining, the Trypan Blue method of Koske and Gemma (1989) was used. Root samples were collected from 3 soybean and maize plants from each treatment. The roots were washed to remove all soil particles and then fine root fragments of 2 cm in length were taken as samples. Root samples were placed in tissue processing cassettes (Ted Pella Inc., Redding, California, United States), and subsequently placed in 10% KOH at 90 °C for 20 min. Cleaned roots were rinsed 5 times with water to remove KOH and immersed in 2% HCl at room temperature for 20 min for acidification. The cassettes containing the roots were immediately stained with 0.05% Trypan Blue (Sigma-Aldrich, St. Louis, Missouri, United States) by overnight incubation at room temperature (∼22 °C). After staining, the roots were transferred to flasks containing lactoglycerol solution and stored at 4 °C to avoid discoloration.

The stained root samples were stored in lactoglycerol for 48 h before mounting the slides. To mount the slides, 10 microscope slides measuring 2 cm in length were arranged in parallel on each microscope slide and covered with a cover slip using lactoglycerol as the mounting medium to preserve the samples. To quantify fungal structures in soybean and maize roots, the numbers of arbuscules and vesicles were counted for each fragment across 10 slides per treatment (totaling 100 subsamples per treatment, with individual plants as the experimental units) using a compound microscope (Olympus, CH2, Tokyo, Japan) at 40× magnification. Root fragments containing other fungal structures such as hyphae and spores were also considered colonized by the fungus. The identification of *R. irregularis* was based on morphological characteristics of spores and hyphae observed under the microscope, consistent with descriptions from the literature. Neither soil nor seeds were sterilized prior to the experiment.

### Statistical Analysis

The data were tested for normality of residuals and homogeneity of variances using the Kolmogorov–Smirnov and Levene tests, respectively. All greenhouse experiments were conducted under a completely randomized factorial design (2 × 2), with the factors AMF inoculation (absence or presence) and Si addition (absence or presence), totaling 4 treatment combinations and 15 replicates (pots) per combination for both soybean and maize.

Plant growth variables measured over time (eg number of trifoliate leaves and plant height) were analyzed using factorial linear mixed-effects models, with AMF, Si, sampling time (DAP), and their interactions included as fixed effects, and plant included as a random factor to account for repeated measurements over time.

Final biomass variables (root and shoot dry weight), measured destructively at 45 DAP, were analyzed using 2-way ANOVA under a completely randomized design, including AMF, Si, and their interaction as fixed factors.

Mycorrhizal colonization data were analyzed considering only AMF-inoculated treatments (AMF-G and AMF-G + Si). Comparisons between Si addition levels within inoculated plants were performed using Student’s *t*-test at a 5% significance level.

When significant effects were detected, mean comparisons were interpreted based on the factorial structure of the model. All statistical analyses were performed using SAS software version 9.4 (SAS Institute 2013).

## Results

### Effects of Si and AMF-G on Plant Growth and Development

In uninjured soybean plants, mean values of TL and PH increased markedly from 30 to 45 DAP across all treatments (Table 1). At 30 DAP, AMF-inoculated plants showed higher mean TL and PH compared to noninoculated plants. At 45 DAP, AMF-G plants also presented the highest mean values for both variables; however, the factorial analysis did not detect a significant main effect of AMF for TL or PH (Table 2). Si alone did not show a consistent increase in growth compared to the control (Table 1).

**Table 1.** Mean (± SE) numbers of trifoliate leaves (TL) and plant heights (PHs) of uninjured soybean plants at 30 and 45 d after planting (DAP), and root dry weights (R) and shoot dry weights (S) of uninjured soybean plants at 45 DAP

Treatments	30 DAP		45 DAP		Dry weight (45 DAP)	
	TL	PH (cm)	TL	PH (cm)	R (g)	S (g)
**C**	5.0 ± 0.2	46.6 ± 3.63	10.8 ± 0.5	120.7 ± 4.4	0.70 ± 0.05	3.63 ± 0.30
**Si**	4.6 ± 0.2	40.1 ± 2.53	9.3 ± 0.6	107.5 ± 6.5	0.68 ± 0.06	2.80 ± 0.28
**AMF-G**	6.2 ± 0.3	55.2 ± 2.36	12.5 ± 0.7	126.5 ± 3.9	0.97 ± 0.03	5.13 ± 0.26
**AMF-G+Si**	5.9 ± 0.1	52.0 ± 1.55	10.1 ± 0.5	125.7 ± 4.0	0.92 ± 0.05	4.61 ± 0.27

Treatments refer to soil treatments: (i) control (C) (no soil amendment); (ii) silicon (Si); (iii) *Rhizophagus irregularis* granular inoculum (AMF-G); (iv) AMF-G + Si.

**Table 2.** Results of factorial linear mixed-effects models from Table 1 with AMF and Si as fixed factors and plant as a random factor

Effect	TL	PH (cm)	R (g)	S (g)
**AMF**	*F* (1,68) = 0.59	*F* (1,71) = 0.47	*F* (1,56) = 28.23	*F* (1,56) = 34.98
	*P* = 0.446^ns^	*P* = 0.495^ns^	*P* < 0.001	P < 0.001
**SI**	*F* (1,68) = 4.06	*F* (1,71) = 0.02	*F* (1,56) = 0.36	*F* (1,56) = 5.81
	*P* = 0.048	*P* = 0.965^ns^	*P* = 0.548^ns^	P = 0.019
**DAP**	*F* (1,56) = 542.07	*F* (1,56) = 1,488.61	—	—
	*P* < 0.001	*P* < 0.001	—	—
**AMF×SI**	*F* (1,68) = 1.22	*F* (1,71) = 0.59	*F* (1,56) = 0.07	*F* (1,56) = 0.31
	*P* = 0.272^ns^	*P* = 0.446^ns^	*P* = 0.798^ns^	*P* = 0.579^ns^
**AMF×DAP**	*F* (1,56) = 0.14	*F* (1,56) = 0.22	—	—
	*P* = 0.709^ns^	*P* = 0.642^ns^	—	—
**SI×DAP**	*F* (1,56) = 9.42	*F* (1,56) = 0.35	—	—
	*P* = 0.003	*P* = 0.556^ns^	—	—
**AMF×SI×DAP**	*F* (1,56) = 2.02	*F* (1,56) = 1.54	—	—
	*P* = 0.160^ns^	*P* = 0.220^ns^	—	—

**Abbreviation: ns, :**not significant.

For biomass measured at 45 DAP, AMF-inoculated plants exhibited higher mean root and shoot dry weights than noninoculated plants. Si alone resulted in similar root biomass compared to the control and slightly lower shoot biomass. The combined AMF-G + Si treatment showed intermediate to high biomass values (Table 1).

Factorial analysis confirmed these patterns (Table 2). The number of TL was strongly affected by sampling time (DAP; *F* (1,56)=542.07, *P* < 0.001). A significant main effect of Si was detected (*F* (1,68)=4.06, *P* = 0.048), as well as a significant Si × DAP interaction (*F* (1,56)=9.42, *P* = 0.003), indicating that Si effects varied across plant developmental stages. No significant AMF × Si interaction was observed for TL.

PH was also strongly influenced by DAP (*F* (1,56)=1488.61, *P* < 0.001), but no significant effects of AMF, Si, or their interaction were detected, indicating that apparent differences among treatments in Table 1 reflect temporal growth dynamics rather than consistent treatment effects.

Root and shoot dry weights measured at 45 DAP were strongly influenced by AMF inoculation. AMF significantly increased root biomass (*F* (1,56)=28.23, *P* < 0.001) and shoot biomass (*F* (1,56)=34.98, *P* < 0.001). Si had no effect on root dry weight (*P* = 0.548), but significantly increased shoot biomass (*F* (1,56)=5.81, *P* = 0.019). No significant AMF × Si interaction was detected for either biomass variable, indicating additive rather than synergistic effects.

In uninjured maize plants, mean values of NL and PH were similar among soil treatments at 15 DAP (Table 3). At 30 DAP, leaf number remained relatively similar across treatments, whereas plant height was greater in AMF-inoculated plants compared to noninoculated plants, consistent with the significant AMF effect detected in the factorial analysis (Table 4).

**Table 3.** Mean (± SE) numbers of leaves (NL), plant heights (PHs), root dry weights (R), and shoot dry weights (S) of undamaged maize plants at 15 and 30 d after planting (DAP)

Treatments	15 DAP		30 DAP		Dry weight (30 DAP)	
	NL	PH (cm)	NL	PH (cm)	R (g)	S (g)
**C**	4.1 ± 0.1	56.7 ± 1.3	7.1 ± 0.1	91.9 ± 2.0	0.56 ± 0.04	1.84 ± 0.16
**Si**	4.4 ± 0.2	60.8 ± 1.7	7.3 ± 0.1	95.7 ± 1.5	0.82 ± 0.08	2.00 ± 0.12
**AMF-G**	4.4 ± 0.2	53.4 ± 1.5	7.5 ± 0.2	102.2 ± 2.5	0.65 ± 0.06	2.03 ± 0.11
**AMF-G+Si**	4.5 ± 0.2	54.0 ± 1.8	7.0 ± 0.1	99.8 ± 3.4	0.71 ± 0.06	2.00 ± 0.14

Treatments refer to soil treatments: (i) control (C) (no soil amendment); (ii) silicon (Si); (iii) *Rhizophagus irregularis* granular inoculum (AMF-G); (iv) AMF-G + Si.

**Table 4.** Results of factorial linear mixed-effects models from Table 3 with AMF and Si as fixed factors and plant as a random factor

Effect	NL	PH (cm)	R (g)	S (g)
**AMF**	*F* (1,51) = 1.55	*F* (1,47) = 16.15	*F* (1,36) = 0.01	*F*(1,36) = 0.48
	*P* = 0.219^ns^	*P* < 0.001	*P* = 0.906^ns^	*P* = 0.490^ns^
**SI**	*F* (1,51) = 3.83	*F* (1,47) = 0.86	*F* (1,36) = 7.14	*F*(1,36) = 0.20
	*P* = 0.056^ns^	*P* = 0.357^ns^	*P* = 0.011	*P* = 0.660^ns^
**DAP**	*F* (1,36) = 1155.58	*F* (1,36) = 949.31	—	—
	*P* < 0.001	*P* < 0.001	—	—
**AMF×SI**	*F* (1,51) = 0.28	*F* (1,47) = 0.01	*F* (1,36) = 2.80	*F*(1,36) = 0.55
	*P* = 0.596^ns^	*P* = 0.926^ns^	*P* = 0.103^ns^	*P* = 0.463^ns^
**AMF×DAP**	*F* (1,36) = 0.79	*F* (1,36) = 21.01	—	—
	*P* = 0.381^ns^	*P* < 0.001	—	—
**SI×DAP**	*F* (1,36) = 4.28	*F* (1,36) = 0.38	—	—
	*P *= 0.046	*P* = 0.541^ns^	—	—
**AMF×SI×DAP**	*F* (1,36) = 2.18	*F* (1,36) = 0.25	—	—
	*P* = 0.148^ns^	*P* = 0.616^ns^	—	—

**Abbreviation: ns, :**not significant.

Regarding biomass at 30 DAP, plants grown in Si-amended soil showed higher mean root dry weight, followed by AMF-G + Si and AMF-G alone, while control plants presented the lowest mean values. Shoot dry weight showed little variation among treatments (Table 3).

Factorial analysis confirmed these patterns (Table 4). The number of leaves was strongly affected by sampling time (DAP; *F* (1,36)=1155.58, *P* < 0.001), but not by AMF inoculation or Si addition. However, a significant Si × DAP interaction (*F* (1,36)=4.28, *P* = 0.046) indicated that Si effects varied across developmental stages.

PH was significantly influenced by AMF inoculation (*F* (1,47)=16.15, *P* < 0.001) and sampling time (*F* (1,36)=949.31, *P* < 0.001), with a significant AMF × DAP interaction (*F* (1,36)=21.01, *P* < 0.001), indicating that AMF effects depended on plant age.

For biomass variables, Si significantly increased root dry weight (*F* (1,36)=7.14, *P* = 0.011), whereas shoot dry weight was not affected by AMF, Si, or their interaction.

### Plant Recovery from Defoliation

In soybean plants with previous mechanical damage (removal of 80% of the TL), PH, and root dry weight did not differ among soil treatments (Table 5). However, the number of new trifoliates after mechanical damage differed among treatments, with higher values in the AMF-G + Si treatment than in the control (C) treatment. A difference was also observed in the dry weights of the aerial portions of plants, with a higher biomass of the aerial portion in the AMF-G + Si treatment than in the C and Si treatments. Shoot dry weights in the AMF-G treatment were also higher than in the C treatment. Thus, in general, plants in the treatments that included AMF recovered better from removal of leaves compared to the control with no soil amendment.

**Table 5.** Mean (±SE) numbers of trifoliate leaves (TL), plant heights (PHs), root dry weights (R), and shoot dry weights (S) of soybean plants with mechanical damage at 45 d after planting (DAP) (15 d after mechanical damage)

Treatments	45 DAP		Dry weight (45 DAP)	
	TL	PH (cm)	R (g)	S (g)
**C**	3.9 ± 0.3b	45.7 ± 2.8a	0.42 ± 0.03a	1.13 ± 0.10c
**Si**	5.1 ± 0.5ab	51.3 ± 3.5a	0.49 ± 0.03a	1.36 ± 0.13bc
**AMF-G**	5.1 ± 0.5ab	42.0 ± 1.1a	0.47 ± 0.05a	1.75 ± 0.17ab
**AMF-G+Si**	6.2 ± 0.3a	43.8 ± 1.9a	0.54 ± 0.04a	2.15 ± 0.12a
***F***	4.83 (3,56)	2.57 (3,56)	1.83 (3,56)	11.64 (3,56)
***P***	0.0047	0.06^ns^	0.15^ns^	<0.0001

Treatments refer to soil treatments: (i) control (C) (no soil amendment); (ii) silicon (Si); (iii) *Rhizophagus irregularis* granular inoculum (AMF-G); (iv) AMF-G + Si. Means followed by different lowercase letters in each column differ significantly by Tukey’s test (*P* < 0.05).

**Abbreviation: ns, :**not significant.

In maize plants with previous mechanical injury (entire plant cut at 5 cm from the ground), there were no differences among soil treatments in plant height 15 d after damage. However, numbers of leaves, root dry weights, and shoot dry weights differed among treatments. Mechanically injured plants treated only with Si (wollastonite) had the highest mean root weights at 15 d after damage, and root weights in this treatment were higher than root weights in the 2 treatments that received AMF. In plants to which neither Si or AMF-G had been applied (control), greater weights of stem and leaves was seen than in AMF-G + Si and AMF-G treatments (Table 6). Numbers of leaves in the C and Si treatments were higher than numbers of leaves in the AMF-G treatment.

**Table 6.** Mean (±SE) number of leaves (NL), plant height (PH), root dry weight (R), and shoot dry weight (S) of maize plants subjected to mechanical damage at 30 d after planting (DAP) (15 d after mechanical damage)

Treatments	30 DAP		Dry weight (30 DAP)	
	NL	PH (cm)	R (g)	S (g)
**C**	4.0a	74.5 ± 3.4a	0.29 ± 0.02ab	0.91 ± 0.09a
**Si**	4.0a	74.8 ± 2.2a	0.33 ± 0.03a	0.86 ± 0.05ab
**AMF-G**	3.6 ± 0.2b	69.0 ± 3.8a	0.19 ± 0.02c	0.61 ± 0.07b
**AMF-G+Si**	3.7 ± 0.1ab	69.2 ± 3.8a	0.24 ± 0.02bc	0.69 ± 0.06ab
***F***	3.40 (3,56)	0.90 (3,56)	6.43 (3,56)	4.13 (3,56)
***P***	0.0280	0.45^ns^	0.0013	0.0129

Treatments refer to soil treatments: (i) control (C) (no soil amendment); (ii) silicon (Si); (iii) *Rhizophagus irregularis* granular inoculum (AMF-G); (iv) AMF-G + Si. Means followed by different lowercase letters in each column differ significantly by Tukey’s test (*P* < 0.05).

**Abbreviation: ns, :**not significant.

### *Spodoptera frugiperda* Growth and Development

Regarding the development of *S. frugiperda* fed on soybean plants, there were no differences among treatments in WG, and RGR of caterpillars. However, caterpillars fed on plants that were treated with the mixture of Si and mycorrhiza showed numerically lower means for WG and RGR. Furthermore, plants in the AMF-G + Si treatment were significantly less consumed by caterpillars after 6 d, followed in ascending order by AMF-G, Si, and the control with the highest percentage of defoliation by *S. frugiperda* (Table 7).

**Table 7.** Mean (±SE) weight gains (WG), relative growth rates (RGR) of caterpillars (*Spodoptera frugiperda*) and percent defoliation (D) of soybean plants infested with *S. frugiperda*

Treatments	WG (mg)	RGR (mg/mg/d)	D (%)
**C**	267.65 ± 26.41a	0.3084 ± 0.0029a	41 ± 3a
**Si**	262.99 ± 20.20a	0.3094 ± 0.0021a	32 ± 3ab
**AMF-G**	311.54 ± 21.09a	0.3143 ± 0.0011a	30 ± 2bc
**AMF-G+Si**	228.76 ± 22.47a	0.3017 ± 0.0056a	19 ± 3c
***F***	2.25 (3,56)	2.29 (3,56)	9.13 (3,56)
***P***	0.09^ns^	0.09^ns^	<0.0001

Treatments refer to soil treatments: (i) control (C) (no soil amendment); (ii) silicon (Si); (iii) *Rhizophagus irregularis* granular inoculum (AMF-G); (iv) AMF-G + Si. Means followed by different lowercase letters in the column differ significantly by Tukey’s test (*P* < 0.05).

**Abbreviation: ns, :**not significant.

For the experiment in which *S. frugiperda* were fed maize plants grown under different soil treatments, there were no differences among soil treatments in WG, RGR of caterpillars, or percentage of defoliation. However, the caterpillars that were fed plants that were treated with Si individually or together with the arbuscular mycorrhizal fungus had the lowest numerical means, respectively, of WG (149.43 and 153.38 mg), and RGR (0.2969 and 0.2963 mg/mg/day). In addition, the plants that received Si showed the lowest average leaf area consumed, with 45.71% defoliation (Table 8).

**Table 8.** Mean (±SE) of weight gain (WG), relative growth rate (RGR) of caterpillars (*Spodoptera frugiperda*) and percentage of defoliation (D) of maize plants infested with *S. frugiperda*

Treatments	WG (mg)	RGR (mg/mg/d)	D (%)
**C**	168.48 ± 12.35	0.3005 ± 0.0023	51 ± 6
**Si**	149.43 ± 10.38	0.2969 ± 0.0017	46 ± 4
**AMF-G**	157.07 ± 15.05	0.2977 ± 0.0026	63 ± 7
**AMF-G+Si**	153.38 ± 12.65	0.2963 ± 0.0029	65 ± 6
***F***	0.39 (3,56)	0.53 (3,56)	2.36 (3,56)
***P***	0.76^ns^	0.66^ns^	0.08^ns^

Treatments refer to soil treatments: (i) control (C) (no soil amendment); (ii) silicon (Si); (iii) *Rhizophagus irregularis* granular inoculum (AMF-G); (iv) AMF-G + Si.

**Abbreviation: ns, :**not significant.

### Quantification of Mycorrhizal Colonization in the Roots

To confirm that soybean and maize roots treated with the AMF-G inoculum were colonized by *R. irregularis*, fungal structures (arbuscules and vesicles) associated with the roots were quantified under a microscope. No fungal structures were observed in plants that did not receive the inoculum (C and Si treatments).

In contrast, roots of soybean and maize plants inoculated with *R. irregularis* (AMF-G and AMF-G + Si) showed the presence of both arbuscules and vesicles (Table 9). For soybean, comparisons between AMF-inoculated treatments revealed that the number of arbuscules was significantly higher in plants receiving AMF-G + Si than in those receiving AMF-G alone (*T *= −2.06, *P* = 0.040), whereas vesicle abundance did not differ between treatments (*P* = 0.653).

**Table 9.** Mean (±SE) of arbuscules and vesicles in maize and soybean roots

Treatments	Maize		Soybean	
	Arbuscules	Vesicles	Arbuscules	Vesicles
**AMF-G**	240 ± 49.3	20.9 ± 5.47	289 ± 47.3	49.9 ± 7.14
**AMF-G+Si**	228.9 ± 37.1	71.4 ± 12.4	395.3 ± 29	54.3 ± 6.48
***t***	0.18 (71)	−3.76 (71)	−2.06 (198)	−0.45 (198)
***P***	0.85^ns^	<0.001	0.040	0.65^ns^

Treatments refer to soil treatments: (i) *Rhizophagus irregularis* granular inoculum (AMF-G); (ii) AMF-G + Si with results of Student’s *t*-tests comparing the effects of soil treatments on the numbers of arbuscules and vesicles in soybean and maize roots. Values are *t* statistics with degrees of freedom in parentheses. Significance was assessed at *P* < 0.05.

**Abbreviation: ns, :**not significant.

For maize, vesicle abundance was significantly higher in plants treated with AMF-G + Si compared to AMF-G alone (*T *= −3.76, *P* < 0.001), while arbuscule abundance did not differ between treatments (*P* = 0.855).

## Discussion

This study offers new insights into the effects of soil amendments with arbuscular AMF and Si on 2 major crops, soybean and maize, grown under the same experimental conditions. The parallel design of the experiments enabled direct comparison between species and revealed significant differences in their physiological responses to the same treatments. One of the most relevant findings is the greater sensitivity of soybean to AMF inoculation. Inoculation with *R. irregularis* consistently enhanced growth and recovery after defoliation in soybean, whereas maize plants showed weaker or even absent responses to AMF under similar conditions. These results align with previous studies demonstrating increased biomass, root colonization, and physiological resilience in soybean following AMF inoculation (Cely et al. 2016, Zhao et al. 2019, Qin et al. 2020, Ulhoa et al. 2025). In contrast, maize appeared more responsive to Si fertilization, particularly in terms of root biomass accumulation in both undamaged and injured plants. This confirms findings from da Silva et al. (2021) and Semina et al. (2020), who also reported enhanced biomass and yield-related traits in maize following Si application. Notably, although soybean was more responsive to AMF, Si also significantly increased shoot biomass in this crop, indicating that Si effects are not restricted to monocots and that both amendments can contribute to soybean development.

An important limitation of this study concerns the strain identity of the *S. frugiperda* colony used in the bioassays. Because larvae were collected annually from rice fields, the colony likely represents the rice strain (sfR). *S. frugiperda* is well documented to consist of 2 host-associated strains, the corn strain (sfC) and the rice strain (sfR), which differ in host preference and performance (Pashley 1986). Since maize is preferentially attacked by sfC under field conditions, the use of sfR in our maize bioassays may have reduced the ecological relevance of the results obtained for this crop. Consequently, the maize-related findings should be interpreted as preliminary and strain-specific, and future studies should include both strains to fully assess the effects of Si and AMF on maize resistance to *S. frugiperda*.

A second aspect of this work is the evaluation of AMF and Si in combination. The factorial analysis did not detect significant AMF × Si interactions for biomass variables in either crop, indicating that the effects of these amendments were predominantly additive under our experimental conditions. However, for some variables in soybean, mean values in the combined AMF-G + Si treatment were numerically lower than those of AMF-G alone, suggesting a possible trend toward reduced performance when both amendments are applied together. Although these numerical patterns should be interpreted with caution given the lack of statistical significance, they are worth noting because most studies report synergistic or neutral interactions between biofertilizers and mineral amendments (eg Abdel-Latef et al. 2016, Moradtalab et al. 2019). If confirmed by future studies, possible mechanisms could include nutrient imbalance caused by excess calcium from the wollastonite source or competition for carbon resources between the mycorrhizal symbiont and Si-mediated defense pathways (Begum et al. 2019). Interestingly, mycorrhizal colonization data showed that Si addition increased arbuscule abundance in soybean and vesicle abundance in maize (Table 9), suggesting that Si did not impair fungal colonization per se. This apparent disconnect between enhanced colonization and reduced plant performance in the combined treatment may indicate that greater colonization does not necessarily translate into proportionally greater functional benefits, particularly when the carbon cost of maintaining the symbiosis competes with other Si-induced physiological demands. These observations underscore the need for further studies on the compatibility between AMF inoculants and mineral Si sources.

With regard to herbivore resistance, another key observation was the reduction in defoliation of soybean plants treated with both AMF and Si. Although effects on *S. frugiperda* larval performance were not statistically significant, lower defoliation levels suggest the presence of induced antixenosis-type resistance, as the reduced feeding was associated with soil treatments rather than constitutive plant traits. The mechanisms underlying this may include increased Si accumulation, which leads to deposition of amorphous silica in leaf tissues creating a physical barrier that reduces palatability and increases mandibular wear in herbivores (Goussain et al. 2002, Massey and Hartley 2009), and secondary metabolite modulation induced by AMF colonization. AMF can upregulate the jasmonic acid signaling pathway, which in turn increases the production of defensive compounds such as phenolics and terpenoids that reduce herbivore preference (Copetta et al. 2006, Babikova et al. 2014, Shrivastava et al. 2015). Previous work has shown that Si can reduce leaf palatability (Massey and Hartley 2009) and damage insect mandibles (Goussain et al. 2002), and that AMF may increase uptake of Si and modulate defense pathways (Walker et al. 2012, Shrivastava et al. 2015). Thus, even though larval weights did not vary significantly, the reduced feeding damage supports the potential of AMF and Si combinations to enhance resistance traits in soybean.

In maize, Si application resulted in numerically lower defoliation levels, but neither larval performance nor defoliation differed significantly among treatments. Therefore, no detectable resistance, whether antixenosis or antibiosis, was observed in maize under the experimental conditions of this study. It should be noted that the use of the rice strain (sfR) in these assays may have influenced these results, as the corn strain (sfC) may respond differently to Si-induced plant defenses. The numerical trends observed are consistent with previous findings of Si-mediated effects in maize through both mechanical reinforcement of tissues and chemical changes, such as volatile emission (Peixoto et al. 2011, Baldin et al. 2019, Ulhoa et al. 2020). However, unlike soybean, no synergistic effect of AMF and Si was detected in maize, and caterpillar performance remained largely unchanged across treatments. This suggests that maize may be less responsive overall to these soil amendments in terms of induced resistance.

Lastly, this study also contributes data on plant tolerance after mechanical injury. It should be noted that mechanical clipping, while useful for standardizing the level of tissue removal, does not fully replicate the feeding behavior of *S. frugiperda*, which causes gradual, localized damage and may induce different plant defense responses. Therefore, the tolerance results presented here should be interpreted with this caveat in mind. With this consideration, AMF improved tolerance in soybean, as shown by faster recovery and greater shoot biomass, while Si improved root regrowth in maize. Interestingly, AMF seemed to suppress postinjury recovery in maize. This unexpected result has not been frequently reported and warrants further investigation, particularly under field conditions with real herbivory and a broader range of AMF species or crop genotypes. The rationale for including mechanical damage experiments alongside caterpillar feeding assays was to separate the effects of tissue loss per se from the specific plant–herbivore interaction. Mechanical damage allows standardization of the amount of tissue removed, enabling a clearer assessment of the plant’s compensatory capacity (tolerance), whereas caterpillar feeding integrates both the plant’s ability to deter feeding (resistance) and its recovery response.

This study highlights the differential roles of Si and AMF in improving crop development, tolerance, and resistance to herbivory, and shows that crop species respond differently to the same soil amendments. These species-specific responses likely reflect differences in root architecture, mycorrhizal dependency, and Si uptake capacity. Soybean, as a dicotyledon with relatively high mycorrhizal dependency, benefited more from AMF colonization, which enhanced nutrient uptake and overall plant vigor. In contrast, maize, a monocotyledon with active Si transporters (Lsi1 and Lsi2), was more responsive to Si application, which enhanced root development and contributed to physical reinforcement of leaf tissues. Soybean benefited more from AMF inoculation, whereas maize responded better to Si application. Additionally, although the factorial analysis indicated predominantly additive effects, numerical trends in soybean suggest that the combination of Si and AMF may not always yield additional benefits over AMF alone, an observation that deserves further investigation.

The reduction in foliar damage caused by *S. frugiperda* in soybean treated with AMF and Si suggests a promising avenue for integrated pest management. While larval development was not significantly affected, the observed antixenosis reinforces the value of using soil amendments as tools to enhance resistance.

Given the growing concerns about insecticide resistance and environmental impact, these low-cost and sustainable alternatives, especially when tailored to crop-specific responses, may be key in building resilient agroecosystems. Preliminary field results have already demonstrated that AMF and Si applications benefit soybean growth and development under field conditions (Ulhoa et al. 2025), supporting the practical relevance of the greenhouse findings presented here. However, it should be noted that the maize-related results are preliminary and strain-specific, as the *S. frugiperda* colony used likely represented the rice strain (sfR) rather than the corn strain (sfC), which is the predominant strain attacking maize under field conditions. Future work should focus on field validation, explore different genotypes and both *S. frugiperda* strains, and further investigate the physiological basis of the AMF and Si interaction.

## References

[ieag028-B1] Abdel-Latef A , Abdel-Rahman H, Hashem A, et al 2016. Arbuscular mycorrhizal symbiosis and abiotic stress in plants. J. Plant Biol. 59:407–426. 10.1007/s12374-016-0237-7

[ieag028-B2] Alhousari F , Greger M. 2018. Silicon and mechanisms of plant resistance to insect pests. Plants (Basel) 7:33. 10.3390/plants7020033 29652790 PMC6027389

[ieag028-B3] Babikova Z , Gilbert L, Bruce T, et al 2014. Arbuscular mycorrhizal fungi and aphids interact by changing host plant quality and volatile emission. Funct. Ecol. 28:375–385. 10.1111/1365-2435.12181

[ieag028-B4] Baldin ELL , Vendramim JD, Lourenção AL. 2019. Resistência de plantas a insetos: fundamentos e aplicações. FEALQ.

[ieag028-B5] Barber NA , Kiers ET, Theis N, et al 2013. Linking agricultural practices, mycorrhizal fungi, and traits mediating plant-insect interactions. Ecol. Appl. 23:1519–1530. 10.1890/13-0156.1 24261037

[ieag028-B6] Barros EM , Torres J, Bianco B, et al 2010. Ovipositiondevelopment and reproduction f *Spodoptera frugiperda* (J.E. Smith) (Lepidoptera: Noctuidae) fed on different hosts of economic importance. Neotrop. Entomol. 39:996–1001. 10.1590/s1519-566x2010000600023 21271070

[ieag028-B7] Begum N , Qin C, Ahanger MA, et al 2019. Role of arbuscular mycorrhizal fungi in plant growth regulation: implications in abiotic stress tolerance. Front. Plant Sci. 10:1068. 10.3389/fpls.2019.01068 31608075 PMC6761482

[ieag028-B8] Bueno AF , Carvalho GA, Santos AC, et al 2017. Pesticide selectivity to natural enemies: challenges and constraints for research and field recommendation. Cienc. Rural 47:1-10. 10.1590/0103-8478cr20160829

[ieag028-B9] Campos-Soriano L , García-Martínez J, San Segundo B. 2012. The arbuscular mycorrhizal symbiosis promotes the systemic induction of regulatory defence-related genes in rice leaves and confers resistance to pathogen infection. Mol. Plant Pathol. 13:579–592. 10.1111/j.1364-3703.2011.00773.x 22212404 PMC6638712

[ieag028-B10] Cely MVT , De Oliveira AG, De Freitas VF, et al 2016. Inoculant of arbuscular mycorrhizal fungi (*Rhizophagus clarus*) increase yield of soybean and cotton under field conditions. Front. Microbiol. 7:720. 10.3389/fmicb.2016.00720 27303367 PMC4880672

[ieag028-B11] Chaudhary G , Singh SK. 2019. Global status of genetically modified crops and its commercialization. In: Khoobchandani M, Saxena A, editors. Biotechnology products in everyday life. Springer International Publishing. p. 147–160.

[ieag028-B12] Copetta A , Lingua G, Berta G. 2006. Effects of three AM fungi on growth, distribution of glandular hairs, and essential oil production in *Ocimum basilicum* L. var. Genovese. Mycorrhiza 16:485–494. 10.1007/s00572-006-0065-6 16896796

[ieag028-B13] Currie AF , Murray PJ, Gange AC. 2011. Is a specialist root-feeding insect affected by arbuscular mycorrhizal fungi? Appl. Soil Ecol. 47:77–83. 10.1016/j.apsoil.2010.12.002

[ieag028-B14] da Silva ES , Prado RM, Soares AAVL, et al 2021. Response of maize seedlings (*Zea mays* L.) to different concentrations of nitrogen in absence and presence of silicon. Silicon 13:813–818. 10.1007/s12633-020-00480-8

[ieag028-B15] Debona D , Rodrigues FA, Datnoff LE. 2017. Silicon’s role in abiotic and biotic plant stresses. Annu. Rev. Phytopathol. 55:85–107. 10.1146/annurev-phyto-080516-035312 28504920

[ieag028-B16] Eghrari K , Oliveira SC, Nascimento AM, et al 2022. The implications of homozygous vip3Aa20-and cry1Ab-maize on *Spodoptera frugiperda* control. J. Pest Sci. 95:115–127. 10.1007/s10340-021-01362-7

[ieag028-B17] Gange AC. 2001. Species-specific responses of a root- and shoot-feeding insect to arbuscular mycorrhizal colonization of its host plant. New Phytol. 150:611–618. 10.1046/j.1469-8137.2001.00137.x

[ieag028-B18] Geiger F , Bengtsson J, Berendse F, et al 2010. Persistent negative effects of pesticides on biodiversity and biological control potential on European farmland. Basic Appl. Ecol. 11:97–105. 10.1016/j.baae.2009.12.001

[ieag028-B19] Gomes FB , Moraes JC, Santos CD, et al 2005. Resistance induction in wheat plants by silicon and aphids. Sci. Agric. (Piracicaba, Braz) 62:547–551. 10.1590/S0103-90162005000600010

[ieag028-B20] Goussain MM , Moraes JC, Carvalho JG, et al 2002. Efeito da aplicação de silício em plantas de milho no desenvolvimento biológico da lagarta-do-cartucho *Spodoptera frugiperda* (J.E. Smith) (Lepidoptera: Noctuidae). Neotrop. Entomol. 31:305–310. 10.1590/S1519-566X2002000200019

[ieag028-B21] Gutiérrez-Moreno R , Mota-Sanchez D, Blanco CA, et al 2019. Field-evolved resistance of the fall armyworm (Lepidoptera: Noctuidae) to synthetic insecticides in Puerto Rico and Mexico. J. Econ. Entomol. 112:792–802. 10.1093/jee/toy372 30535077

[ieag028-B22] Higo M , Tatewaki Y, Gunji K, et al 2019. Cover cropping can be a stronger determinant than host crop identity for arbuscular mycorrhizal fungal communities colonizing maize and soybean. PeerJ. 7:e6403. 10.7717/peerj.6403 30775179 PMC6369830

[ieag028-B23] Hoffmann-Campo CB , Corrêa-Ferreira BS, Moscardi F. 2012. Soja: manejo integrado de insetos e outros artrópodes praga. Embrapa.

[ieag028-B24] Hohnjec N , Vieweg MF, Puhler A, et al 2005. Overlaps in the transcriptional profiles of *Medicago truncatula* roots inoculated with two different *Glomus* fungi provide insights into the genetic program activated during arbuscular mycorrhiza. Plant Physiol. 137:1283–1301. 10.1104/pp.104.056572 15778460 PMC1088321

[ieag028-B25] Kapoor R , Chaudhary V, Bhatnagar AK. 2007. Effects of arbuscular mycorrhiza and phosphorus application on artemisinin concentration in *Artemisia annua* L. Mycorrhiza 17:581–587. 10.1007/s00572-007-0135-4 17578608

[ieag028-B26] Khaosaad T , Vierheilig H, Nell M, et al 2006. Arbuscular mycorrhiza alter the concentration of essential oils in oregano (*Origanum* sp., Lamiaceae). Mycorrhiza 16:443–446. 10.1007/s00572-006-0062-9 16909287

[ieag028-B27] Koske RE , Gemma JN. 1989. A modified procedure for staining roots to detect VA mycorrhizas. Mycol. Res. 92:486–488. 10.1016/S0953-7562(89)80195-9

[ieag028-B28] Kvedaras OL , An M, Choi Y, et al 2010. Silicon enhances natural enemy attraction and biological control through induced plant defences. Bull. Entomol. Res. 100:367–371. 10.1017/S0007485309990265 19737442

[ieag028-B29] Li Z. 2018. Evaluation of regulatory variation and theoretical health risk for pesticide maximum residue limits in food. J. Environ. Manage. 219:153–167. 10.1016/j.jenvman.2018.04.067 29747101

[ieag028-B30] Liang Y , Nikolic M, Bélanger R, et al 2015. Silicon and insect pest resistance. In: Liang Y, Nikolic M, Bélanger R, Gong H, Song A, editors. Silicon in agriculture. Springer. p. 197–207.

[ieag028-B31] Luginbuehl LH , Menard GN, Kurup S, et al 2017. Fatty acids in arbuscular mycorrhizal fungi are synthesized by the host plant. Science 356:1175–1178. 10.1126/science.aan0081 28596311

[ieag028-B32] Ma JF. 2004. Role of silicon in enhancing the resistance of plants to biotic and abiotic stresses. Soil Sci. Plant Nutr. 50:11–18. 10.1080/00380768.2004.10408447

[ieag028-B33] Massey FP , Hartley SE. 2009. Physical defences wear you down: progressive and irreversible impacts of silica on insect herbivores. J. Anim. Ecol. 78:281–291. 10.1111/j.1365-2656.2008.01472.x 18771503

[ieag028-B34] Meena VD , Dotaniya ML, Coumar V, et al 2014. A case for silicon fertilization to improve crop yields in tropical soils. Proc. Natl. Acad. Sci. 84:505–518. 10.1007/s40011-013-0270-y

[ieag028-B35] Miraldo LL , Bernardi O, Horikoshi RJ, et al 2016. Functional dominance of different aged larvae of Bt-resistant *Spodoptera frugiperda* (Lepidoptera: Noctuidae) on transgenic maize expressing Vip3Aa20 protein. Crop Prot. 88:65–71. 10.1016/j.cropro.2016.05.024

[ieag028-B36] Moradtalab N , Hajiboland R, Aliasgharzad N, et al 2019. Silicon and the association with an arbuscular-mycorrhizal fungus (*Rhizophagus clarus*) mitigate the adverse effects of drought stress on strawberry. Agronomy 9:41. 10.3390/agronomy9010041

[ieag028-B37] Nishida T , Katayama N, Izumi N, et al 2010. Arbuscular mycorrhizal fungi species-specifically affect induced plant responses to a spider mite. Popul. Ecol. 52:507–515. 10.1007/s10144-010-0208-7

[ieag028-B38] Overton K , Maino JL, Day R, et al 2021. Global crop impacts, yield losses and action thresholds for fall armyworm (*Spodoptera frugiperda*): a review. Crop Prot. 145:105641. 10.1016/j.cropro.2021.105641

[ieag028-B39] Parisi C , Tillie P, Rodríguez-Cerezo E. 2016. The global pipeline of GM crops out to 2020. Nat. Biotechnol. 34:31–36. 10.1038/nbt.3449 26744975

[ieag028-B40] Pashley DP. 1986. Host-associated genetic differentiation in fall armyworm (Lepidoptera: Noctuidae): a sibling species complex? Ann. Entomol. Soc. Am. 79:898–904. 10.1093/aesa/79.6.898

[ieag028-B41] Paye W , Tubana B, Harrell D, et al 2018. Determination of critical soil silicon levels for rice production in Louisiana using different extraction procedures. Commun. Soil Sci. Plant Anal. 49:2091–2102. 10.1080/00103624.2018.1495731

[ieag028-B42] Peixoto ML , Moraes JC, Silva AA, et al 2011. Effect of silicon on the oviposition preference of *Bemisia tabaci* Biotype B (Genn.) (Hemiptera: Aleyrodidae) on bean (*Phaseolus vulgaris* L.) plants. Cienc. Agrotecnol. 35:478–481. 10.1590/S1413-70542011000300006

[ieag028-B43] Pozo MJ , Azcon-Aguilar C. 2007. Unraveling mycorrhiza-induced resistance. Curr. Opin. Plant Biol. 10:393–398. 10.1016/j.pbi.2007.05.004 17658291

[ieag028-B44] Qin J , Wang H, Cao H, et al 2020. Combined effects of phosphorus and magnesium on mycorrhizal symbiosis through altering metabolism and transport of photosynthates in soybean. Mycorrhiza 30:285–298. 10.1007/s00572-020-00955-x 32296944

[ieag028-B45] Quintela ED , Barbosa FR. 2015. Manual de identificação de insetos e outros invertebrados pragas do feijoeiro. 2nd ed. Embrapa Arroz e Feijão.

[ieag028-B46] Ranger CM , Singh AP, Frantz JM, et al 2009. Influence of silicon on resistance of *Zinnia elegans* to *Myzus persicae* (Hemiptera: Aphididae). Environ. Entomol. 38:129–136. 10.1603/022.038.0116 19791606

[ieag028-B47] Rapparini F , Llusia J, Penuelas J. 2008. Effect of arbuscular mycorrhizal (AM) colonization on terpene emission and content of *Artemisia annua* L. Plant Biol. (Stuttg) 10:108–122. 10.1055/s-2007-964963 18211551

[ieag028-B48] Reynolds OL , Gurr GM, Padula MP, et al 2016. Silicon: potential to promote direct and indirect effects on plant defence against arthropod pests. Front. Plant Sci. 7:744. 10.3389/fpls.2016.00744 27379104 PMC4904004

[ieag028-B49] SAS Institute. 2013. SAS System for Windows, version 9.4. SAS Institute Inc.

[ieag028-B50] Schausberger P , Peneder S, Jurschik S, et al 2012. Mycorrhiza changes plant volatiles to attract spider mite enemies. Funct. Ecol. 26:441–449. 10.1111/j.1365-2435.2011.01947.x

[ieag028-B51] Schoenherr AP , Rizzo E, Jackson N, et al 2019. Mycorrhiza-induced resistance in potato involves priming of defense responses against cabbage looper (Noctuidae: Lepidoptera). Environ. Entomol. 48:370–381. 10.1093/ee/nvy195 30715218

[ieag028-B52] Semina S , Gavryshina I, Zheryakov E, et al 2020. The formation of maize grain yield when using silicon-containing preparations. Sci. Pap. A. Agron. 63:405–413.

[ieag028-B53] Shrivastava G , Ownley BH, Augé RM, et al 2015. Colonization by arbuscular mycorrhizal and endophytic fungi enhanced terpene production in tomato plants and their defense against herbivorous insect. Symbiosis 65:65–74. 10.1007/s13199-015-0319-1

[ieag028-B54] Singh S , Sahoo MR, Acharya GC, et al 2022. Silicon: a potent nutrient in plant defense mechanisms against arthropods. Silicon 14:6493–6505. 10.1007/s12633-021-01427-3

[ieag028-B55] Smith SE , Read DJ. 2008. Mycorrhizal symbiosis. 3rd ed. Academic Press.

[ieag028-B56] Tomczak VV , Schweiger R, Müller C. 2016. Effects of arbuscular mycorrhiza on plant chemistry and the development and behavior of a generalist herbivore. J. Chem. Ecol. 42:1247–1258. 10.1007/s10886-016-0785-9 27787678

[ieag028-B57] Tudi M , Ruan HD, Wang L, et al 2021. Agriculture development, pesticide application and its impact on the environment. Int. J. Environ. Res. Public Health 18:1112. 10.3390/ijerph18031112 33513796 PMC7908628

[ieag028-B58] Ulhoa LA , Barrigossi JAF, Borges M, et al 2020. Differential induction of volatiles in rice plants by two stink bug species influence behaviour of conspecifics and their natural enemy *Telenomus podisi*. Entomol. Exp. Appl. 168:76–90. 10.1111/eea.12869

[ieag028-B59] Ulhoa LA , Boiça Júnior AL, Davis JA, et al 2025. Influence of arbuscular mycorrhizal fungi and silicon on soybean growth and development in the field. J. Sustain. Agric. Environ. 4:1–11. 10.1002/sae2.70120

[ieag028-B60] Valicente FH. 2015. Manejo integrado de pragas na cultura do milho. Embrapa.

[ieag028-B61] Vannette RL , Hunter MD. 2011. Plant defense theory re-examined: nonlinear expectations based on the costs and benefits of resource mutualisms. J. Ecol. 99:66–76. 10.1111/j.1365-2745.2010.01755.x

[ieag028-B62] Walker V , Couillerot O, Von Felten A, et al 2012. Variation of secondary metabolite levels in maize seedling roots induced by inoculation with *Azospirillum*, *Pseudomonas* and *Glomus* consortium under field conditions. Plant Soil 356:151–163. 10.1007/s11104-011-0960-2

[ieag028-B63] Xiao TJ , Yang QS, Ran W, et al 2019. Effect of inoculation with arbuscular mycorrhizal fungus on photosynthesis and antioxidant enzymatic activity of maize under salt stress. Acta Ecol. Sin. 39:295–301. 10.1016/j.chnaes.2018.09.009

[ieag028-B64] Zhao S , Chen A, Chen C, et al 2019. Transcriptomic analysis reveals the possible roles of sugar metabolism and export for positive mycorrhizal growth responses in soybean. Physiol. Plant. 166:712–728. 10.1111/ppl.12847 30288747

